# Altruism and the Link to Pro-social Pandemic Behavior

**DOI:** 10.3389/frhs.2022.871891

**Published:** 2022-07-14

**Authors:** Sebastian Neumann-Böhme, Iryna Sabat, Arthur E. Attema

**Affiliations:** ^1^Hamburg Center for Health Economics, University of Hamburg, Hamburg, Germany; ^2^EsCHER, Erasmus School of Health Policy and Management, Erasmus University Rotterdam, Rotterdam, Netherlands; ^3^Nova School of Business and Economics, Lisbon, Portugal

**Keywords:** altruism, behavior, behavioral economics, COVID-19, health economics, risk perceptions

## Abstract

In the Corona pandemic, especially in the phase before vaccines were available, people's risk of infection with COVID-19 was dependent on the adherence to pandemic behaviors (e. g., wearing masks) of others around them. To explore whether altruistic individuals are more likely to engage in pro-social behaviors to protect others during the pandemic, we use data from the European COVID Survey (ECOS). The data was collected in September 2020 and consisted of a representative sample from seven European countries (*N* = 7,025). Altruism was measured as a deviation from purely self-interested behavior by asking respondents how much they would be willing to donate from an unexpected gain to the equivalent of 1000€. Respondents who were willing to donate more than 0 Euros (68.7%) were treated as altruistic; on average, respondents were willing to donate 11.7% (SD 17.9) of the gain. Controlling for country, sociodemographics, general risk aversion and COVID-specific risk aversion, we find that individuals classified as altruistic were more likely to behave pro-socially. More specifically, we find that altruistic respondents were more likely to wait at home for test results and wear a mask where it is recommended. They would also stay about 1 day longer under quarantine without symptoms after visiting a high-risk country and were less likely to go to a supermarket with COVID symptoms. We find no significant effect for wearing a mask in places where it is mandatory and for inviting more than six people into the house. Furthermore, we find that the subjective risk assessment of COVID-19 also plays a role in these behaviors. Our results support evidence from the literature that suggests that adherence to pro-social pandemic behaviors may be increased if public health officials emphasize the altruistic nature of these behaviors.

## Introduction

The COVID-19 pandemic has increased the impact the behaviors of others have on someone's health. For example, wearing a face mask or a face filtering piece (FFP) has been shown to significantly reduce the risk of infection with COVID-19 in communities (1). Masks work best if everyone wears them, which is a minor nuisance to the individual but benefits everyone in a given space.

Pandemic behaviors, such as mask-wearing and social distancing, can be seen as contributing to a public good. A public good is non-excludable: individual A cannot be excluded from the protection generated by individual B wearing a mask (2). Public goods are also non-rivalrous: the fact that individual A's risk of infection is reduced by social distancing does not diminish this effect for individual B. Therefore, during the pandemic, certain pro-social behaviors (e.g., the correct and consequent use of masks) contribute to a public good, namely a low-risk environment that reduces the risk of a COVID-19 infection. Most of the behaviors that contribute to the low (infection) risk environment have in common that they require some sacrifice or discomfort for the individual to contribute to the low-risk environment. This can mean standing in line outside to follow social distancing rules or carrying around and wearing face masks. Some of these behaviors will benefit others and the individual making a sacrifice, while many will mainly benefit others. A (selfless) concern for the wellbeing of others, as opposed to selfishness, as a principle of action, is defined as *altruism* by the Oxford English Dictionary (3). In (classical) economics [e.g., (4, 5)], altruism is defined as a behavior that benefits others at one's own expense (6) and a deviation from what rational beings ought to do, maximizing their own wealth. Another explanation in the context of altruism and rationality is provided by Andreoni (7), who discusses that a donation to charity can also provide a warm glow in the form of social recognition to the donor, which would be seen as *impure altruism*.

Altruism has been shown to explain pro-social behavior, for example, in the health (8) and the environmental context (9). Applied to the question at hand, Cato et al. (10) showed that people with higher altruistic concerns and sensitivity to shaming were more likely to follow social distancing measures during the COVID-19 pandemic. Meanwhile, Webster et al. (11) reviewed existing evidence to improve adherence to quarantine measures. The authors argued that adherence could be improved if public health officials provide a timely and clear rationale for quarantine and emphasize social norms to encourage altruistic (pandemic) behaviors. Nikolov et al. (12) performed a longitudinal study in the U.S. during the COVID-19 pandemic. They found that demographic characteristics exert the largest influence on social distancing measures and mask-wearing and that individual risk perception and cognitive biases exert a critical role in influencing the decision to adopt social distancing measures.

Alfaro et al. (13) posited that social preferences facilitate the internalization of health externalities by, for example, reducing mobility during a pandemic. They found that mitigation policies matter less in regions that are more altruistic, patient, or exhibit less negative reciprocity. In those regions, mobility fell ahead of lockdowns and remained low after the lifting thereof.

On the effect of pro-social behavior, Campos-Mercade et al. (14) showed that a large majority of people are very reluctant to put others at risk for their personal benefit. They also find that prosociality predicts health behaviors during the pandemic and suggested that the impact of policies on a population may depend on the degree of prosociality. Applied to the German context at the end of 2020, Fang et al. (15) investigated the role of prosociality in reducing the spread of COVID-19 in a nationally representative survey. They reported that higher prosociality is positively related to compliance with recommended public health behaviors. Their results confirmed that voluntary behavioral change due to pro-social motivations could play an important role in the pandemic.

Müller and Rau (16) analyzed whether economic preferences and pre-crisis social responsibility predict social compliance with policy regulations. Their results show that economic preferences are closely related to compliance with policies fighting a crisis. Risk tolerance negatively affected citizens' avoidance of crowds, whereas patience helped them to do so and to stay home. Pre-crisis socially responsible behavior related to fare evasion, turnout and support of vaccination was also positively related to social compliance.

van Hulsen et al. (17) examined the role of intertemporal and social preferences in explaining cooperation in the social dilemma caused by the intelligent lockdown in The Netherlands. Through an online survey, they measured people's considerations of future consequences and of others and found that both were associated with increased compliance with the precautionary measures.

In order to investigate the relationship between altruism and pro-social pandemic behaviors, we aim to investigate the following hypothesis:

I. More altruistic people are more likely to follow pandemic behaviors that benefit others (at their own cost).

We expect that factors such as overall risk aversion and COVID-19-specific risk assessments will play a role in the pandemic behaviors investigated in this study. Nonetheless, similar to the observations that Rabin (18) makes, we believe that people may be willing to contribute more to a public good than can be explained by self-interest. In other words, we expect that people who show a general disposition to altruistic behavior are also more likely to show pandemic behavior that benefits others at their own disadvantage.

## Methods

The data collected for this article was part of a more extensive data collection. The European COVID Survey (ECOS) started in April 2020 and collected representative samples of the population in seven (eight from June 2021) European countries bi-monthly. More details on the ECOS project and its methodology can be found elsewhere (19, 20). It was part of the third ECOS data collection running in September 2020 (08-18.09.2020). As part of the data collection, certain quality assurance measures were applied. Respondents who did not complete the survey (incompletes), who answered the survey multiple times (doubles), and those who answered the survey faster than 1/3 of the median length of interview in their respective country (speeders) were excluded from the sample. These cases consisted of about 50 responses in total. The total sample of the data collection consisted of 7,025 respondents. Income was elicited as a relative position (“Thinking of your household's total monthly income, would you say that your household is able to make ends meet…”) of household income in relation to the monthly costs.

As a measure of altruism, we asked respondents how much they would donate if they unexpectedly received 1,000 Euros (or the currency and purchasing power adjusted equivalent) (29). Everyone willing to donate more than 0 Euros was considered altruistic (to some degree) since this signals a deviation from purely self-interested behavior. Therefore, we generated a binary variable taking the value of 1 if more than 0 Euros were donated and 0 otherwise to indicate Altruism. We considered and tested (see section Regression Analysis) the percentage of the respondents who donated as a continuous measure based on the share of the equivalent of 1,000 Euros donated. Furthermore, we tested a measure of altruism based on the country specific quartiles for the share donated, to generate levels of altruism (no altruism, low, medium and high level). While these measures use more information than the binary relationship, they do not fit with the definition of altruism as a deviation from purely self-interested behavior (donating zero).

When the data for this article was collected in September 2020, no vaccines against COVID-19 were available in the ECOS countries. The European Commission only approved the first vaccine (Comirnaty by BioNTech) in December 2020 (21). Therefore, the primary way to reduce the risk of infection with COVID-19 at the time was social distancing, testing, and wearing masks.

Based on this, we elicited pandemic behaviors on a 1–4 Likert scale ranging from very unlikely (1) to very likely (4) and “do not know” as an opt-out option. We asked respondents to indicate:

- “You got a COVID-19 test due to symptoms; you are waiting for the results. It can take up to 4 days. Would you stay at home (under quarantine)?”
- “Suppose you show some COVID-19 related symptoms (coughing/fever/feeling tired/sneezing), but it could also be a cold. Would you still go to a supermarket?”
- How likely would you be to:
- “Invite more than 6 people to your house for an indoor gathering.”
- “Wear face masks where it is recommended (e.g., large outside gatherings).”
- “Wear face masks where it is mandatory (e.g., public transport, supermarket).”

Similar to Aschwanden et al. (22), we proceeded to recode these preventative behaviors into binary variables taking the value of 1 to indicate that respondents stated they were likely or very likely to engage in this behavior and 0 otherwise, while “do not know” was recoded to missing.

We analyzed these five behaviors individually, employing a logit regression and reporting the odds ratio of being more or less likely to engage in a given behavior. September 2020 was also when many Europeans returned from summer vacations; therefore, we asked respondents how many days they would voluntarily spend in quarantine after visiting a high-risk country if no COVID-19 test was available.

- “Consider you have to travel to a country that has been designated as a risk area because of the number of COVID infections. You have no symptoms and do not take a test at the airport. For how many days would you stay in quarantine if no test was available?”

Respondents were able to indicate if they would spend 0–14 days under quarantine on a slider. This allowed us to investigate if altruistic people would spend more days in quarantine using an OLS regression with the same set of controls as the other five pandemic behaviors.

We then proceeded to create an index of pro-social behavior (iPSB) by adding together the answers on the Likert scale for each behaviour.^1^ We rescaled the days in quarantine after travel to a 1–4 scale to fit other items on the iPSB. With six items the scale ranges from 6 to 24, where 6 indicates a low score of pro-social behavior and 24 a very high score. Cronbach's α = 0.71 with all six items and α = 0.72 when only the five items with the Likert scale would be included (i.e., without rescaling), suggesting that the internal consistency of the iPSB is at least acceptable. The interitem correlation is below 0.50 for all items, except for the two mask items (mandatory and voluntary mask wearing) where it is 0.71, which is to be expected since both test a similar construct. Due to the high degree of correlation between the two mask related items of the iPSB we conducted a principal component analysis (pca) as a sensitivity check for the iPSB. Using pca, we generated a score that combines the six protective behaviors into one scalar by multiplying each response with the respective factor loading and adding them up to one index. We achieved a Kaiser-Meyer-Olkin measure of sampling adequacy of 0.68, suggesting that the data is suitable for pca (23).

Next to our variable of interest (altruism), we used a vector of control variables: age, gender, country, education, and relative household income. We also included the risk preferences based on income by Barsky et al. (24), offering respondents lotteries that can increase/decrease income as a general measure of risk aversion. In a more COVID-specific subjective measure, respondents were asked how they “think and feel about the risks related to the COVID-19 outbreak.” They could indicate their subjective assessment of how they rate the risk of being infected with COVID-19, and the risk COVID-19 poses to their health, their families' health, and the risk to the health of people in their community. The perceived risk could be indicated on a slider ranging from 1 (no risk at all) to 5 (very high risk). For the regression analysis, we recoded these to 0 (no/low/moderate) and 1 (high/very high) risk. To account for the incidence rate of COVID-19, we used the country-specific 7-day average of confirmed COVID-19 cases per million (cpm) on the day each respondent filled out the survey (25). We used a stepwise regression approach, using OLS and adjusting for heteroscedasticity, with three models to investigate the effect of altruism on the iPSB. Model I uses the sociodemographic controls and the cpm, Model II proceeds to add the general risk preferences, and Model III adds the COVID-related subjective risk factors.

## Results

We report the respondents' sociodemographic characteristics by country and in total in Table 1. The data presented here are largely representative of the population in terms of age category, gender, and regional distribution of the respondents in the respective countries. As shown in Table 1, there are deviations from representativeness regarding the education level in some countries. This reflects a difference between the education level reported to the panel agency Dynata and the education level elicited in ECOS. This constitutes a (known) limitation of online survey methods together with problems of ensuring the representativeness of older individuals in online panels, which in the case of ECOS has been problematic in Portugal.

**Table 1:** Participants' sociodemographics compared to national census^a^.

	**DE**		**UK**		**DK**		**NL**		**FR**		**PT**		**IT**		**Total**
N	1,005		1,005		1,000		1,004		1,001		1,001		1,009		7,025
**Gender**	cen.	ECOS	cen.	ECOS	cen.	ECOS	cen.	ECOS	cen.	ECOS	cen.	ECOS	cen.	ECOS	
Male	48.3	48.3	48.6	49.6	49.1	45.8	49.0	48.3	47.6	46.2	47.8	48.0	47.9	47.4	47.6
Female	51.7	51.7	51.4	50.5	50.9	54.2	51.0	51.7	52.4	53.9	52.2	52.1	52.1	52.6	52.4
**Age category**															
18–24	9.7	7.3	12.0	10.5	11.2	8.1	10.8	10.0	11.4	9.2	9.7	10.7	8.6	7.9	9.1
25–34	14.3	13.5	17.1	16.5	14.9	12.6	15.2	15.4	16.2	15.3	18.2	21.0	14.9	14.9	15.6
35–44	17.0	17.7	17.9	18.5	17.8	16.4	18.8	18.0	18.1	18.9	18.5	21.1	19.3	20.3	18.7
45–54	18.0	18.8	17.7	18.3	18.0	19.0	18.7	18.3	17.5	18.1	17.1	18.4	17.7	18.2	18.5
55–64	16.2	16.9	15.1	15.8	16.1	19.3	16.3	17.2	15.5	16.4	14.6	14.6	15.0	14.8	16.4
65+	24.8	25.8	20.2	20.4	22.0	24.6	20.1	21.0	21.4	22.2	21.9	14.3	24.4	23.9	21.7
**Education**															
Low	17.0	11.5	37.0	14.5	19.3	11.7	34.3	28.7	34.0	21.4	59.0	21.9	44.4	26.1	19.4
Middle	60.0	64.6	36.0	41.0	54.0	55.2	42.1	43.4	41.0	44.6	22.0	34.4	41.3	50.8	47.7
High	23.0	23.9	27.0	44.6	26.7	33.1	23.6	27.9	25.0	34.0	19.0	43.8	14.3	23.1	33.0
**Income (make ends meet)**															
Great difficulty		7.0		5.6		7.8		7.7		13.0		6.3		11.0	8.3
Some difficulty		35.8		28.9		31.4		37.7		44.0		26.0		47.2	35.8
Fairly easily		41.8		43.8		41.4		37.4		34.9		57.0		34.6	41.5
Easily		15.4		21.8		19.4		17.3		8.2		10.7		7.2	14.3

*^a^Compared to the national Census data: Spain, 2011, Instituto Nacional de Estadistica (INE); UK, 2011,Office ofNational Statistics (ONS);Netherlands, 2018, Centraal Bureau voor de Statistiek (CBS); Germany, 2011, Federal StatisticalOffice; France, 2011, National Institute of Statistics and Economic Studies (INSEE); Italy, 2018, Italian National Institute of Statistics; Denmark 2017, Statistics Denmark; Portugal, 2011, Instituto Nacional De Estatistica (INE)/Statistics Portugal*.

### Altruism as a Deviation From Self-Interested Behavior

**Figure 1:**
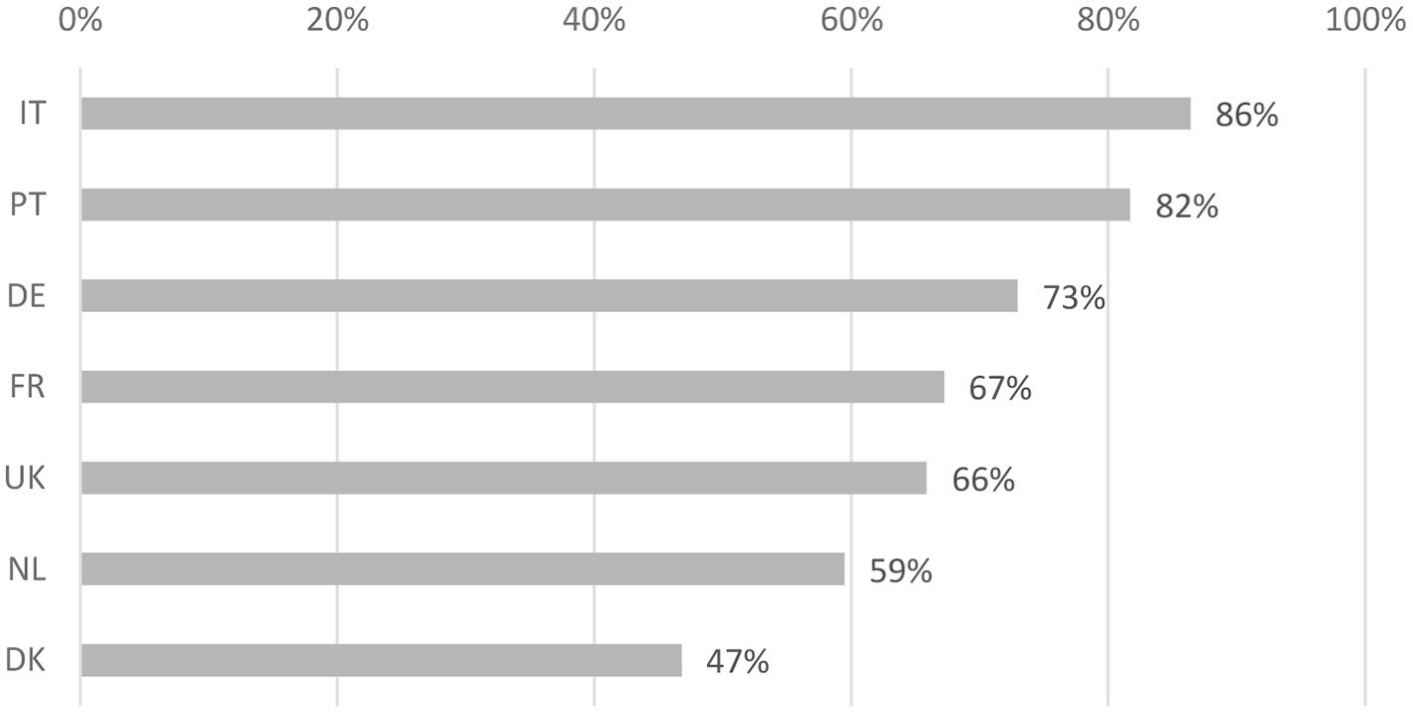
Share of respondents by country, who were willing to donate more than 0 Euro of an unexpected monetary gain of the equivalent of 1,000 Euros.

Overall, 68.65% of respondents were willing to donate more than 0 Euros, with clear differences between countries as visualized in Figure 1. We find that the largest share of respondents willing to donate a share of an unexpected monetary gain was observed in Italy (86%) and the lowest share in Denmark (47%). We find that the willingness to donate is highest among respondents aged 18–24 (75.9%), then decreases gradually to 65.6% among those between the age of 55–64, with those above the age of 65 having the second-highest share (71.8%). As expected, the share of respondents willing to donate is slightly higher (71.8%) among those who report that they get by with their income fairly easy than among those who state to get by with some difficulty (67.5%).

On average, people were willing to donate 11.7% (SD 17.9) of the gain, with a median of 5%.

As outlined above, we generated a measure of altruism or level based on the country specific quartiles of the share donated. We observe a relatively even distribution across levels, with a donation level in the 1st quartile (36%) i.e., no altruism a bit overrepresented and a low level of altruism (2nd quartile, 20%), medium level (3rd quartile, 23%), and high level (4th quartile 21%) more even distributed. We proceeded to test the continuous and ordinal measure as alternative specifications in the regression model.

### Preventative Behaviors

In the figures in the Appendix, we show the descriptive results of each preventative behavior by country. Some of these behaviors were affected by regulations (see Table 2), for example inviting more than six people to the own house, which was affected by the restrictions on gatherings in September 2020. With 37%, the highest likelihood was reported in Italy, where the restrictions on gatherings in September 2020 were not as strict as for example the UK (likelihood 17%). Other behaviors such as waiting for a test result with symptoms or going to a supermarket with COVID symptoms was not directly affected by regulations.

**Table 2:** Restriction level by country.

**CN**	**Gatherings of people limited to**	**Lockdown**	**Restriction to movement**	**Masks**
DE	10 or less	No measure	No measure	Required in **some** specified shared/public spaces
DK	Up to 100	Recommended not to leave house	No measure	Required in **some** specified shared/public spaces
FR	10 or less	No measure	Recommend not to travel between regions/cities	Required outside the home at all times
UK	10 or less	Recommended not to leave house	Internal movement restrictions in place	Required in **all** shared/public spaces
IT	Up to 1,000	Required not leaving house with exceptions for daily exercise, grocery shopping, and 'essential' trips	Internal movement restrictions in place	Required outside the home at **all** times
NL	10 or less	Recommended not to leave house	No measure	Required in **some** specified shared/public spaces
PT	10 or less	No measure	No measure	Required in **some** specified shared/public spaces

### Measures of Risk Aversion

When analyzing the general (income-based) risk aversion (24), by asking respondents two consecutive questions if they would take a gamble to increase or reduce their income,^2^ we find that a majority of the sample is very risk-averse (52%) and that there are differences between the countries in the survey, as shown in Table 3. We find a significant difference in the level of risk aversion among respondents who are altruistic (M = 2.76, SD = 1.30) and those who are not altruistic (M = 3.21, SD = 1.14), *t*_(7, 023)_ = 14.08, *p* = 0.000, indicating a higher level of risk aversion among respondents who are not altruistic.

**Table 3:** General risk aversion.

**Group**	**DE (%)**	**UK (%)**	**DK (%)**	**NL (%)**	**FR (%)**	**PT (%)**	**IT (%)**	**Total (%)**
IV (least risk averse)	23	25	15	17	25	36	30	24
III	13	11	12	12	11	14	16	13
II	11	12	11	12	11	5	13	11
I (most risk averse)	52	51	62	58	53	45	41	52

There is a clear difference in risk preferences between the two southern European countries, which are less risk-averse than their northern neighbors, with France in the middle. A similar distinction between the northern and southern European countries was identified by Sabat et al. (20) when analyzing the economic worries of respondents in the first wave of the ECOS in April 2020.

As indicated in Table 4, we also observe variation in the COVID-related risk factors between countries. Focusing on the different domains of subjective risk perceptions (all countries taken together, row total Table 4), we find a significant difference in means between the perceived risk of infection (M = 2.96, sd = 0.92) and the risk to the own health (M = 3.03, sd = 1.11); *t*_(7, 024)_ = [−6.85], *p* < 0.000, using a paired-samples *t*-test. This could be interpreted as respondents being less worried about the risk of infection than the risks to their health if they would get the disease. Using the same test, we also find a significant difference in means between the perceived risk to the family (M = 3.26, sd = 1.12) and the risk to the community (M = 3.15, sd = 1.02); *t*_(7, 024)_ = [10.59], *p* < 0.000.

**Table 4:** Subjective assessments of risk COVID-19 poses to respondents by country.

	**Risk of infection**		**Risk to own health**		**Risk to family**		**Risk to community**	
**Country**	**Mean**	**Std. dev**.	**Mean**	**Std. dev**.	**Mean**	**Std. dev**.	**Mean**	**Std. dev**.
DE	2.89	0.99	2.99	1.15	3.04	1.15	2.95	1.02
UK	2.97	0.91	3.1	1.07	3.27	1.12	3.22	0.98
DK	2.85	0.85	2.94	1.12	3.17	1.15	2.98	0.97
NL	2.91	0.94	2.93	1.12	3.07	1.12	2.95	1.04
FR	3.11	0.91	3.23	1.09	3.45	1.07	3.33	1.05
PT	2.99	0.89	3.05	1.12	3.51	1.08	3.31	0.97
IT	2.97	0.93	2.99	1.04	3.29	1.05	3.29	0.98
Total	2.96	0.92	3.03	1.11	3.26	1.12	3.15	1.02

### Regression Analysis

We proceed by presenting the regression results of the determinants and characteristics associated, first with the iPSB and afterwards with the pandemic behaviors individually.

#### Index of Pro-social Behavior (IPSB)

Using the index of pro-social behavior (iPSB) that combines the six protective or pro-social behaviors, we find that respondents' average score on the index was 19.5 (SD 3.4, median 20). When looking at the determinants, we find that age (*p* < 0.001) and being female (*p* < 0.001), as well as a medium (*p* < 0.001) or high level of formal education (*p* < 0.01) are significantly associated with a higher iPSB score throughout models. A higher incidence of COVID-19 cases (*p* < 0.05 in Model I, *p* < 0.01 in Model II/III) were positively associated with a higher score, similarly higher levels of income (*p* < 0.05) had a positive association, but it was not consistently significant throughout models, as shown in Table 5. A higher general risk aversion (*p* < 0.001) had a significant positive association with the iPSB.

**Table 5:** Regression results index of pro-social behavior (iPSB).

**iPSB**	**Model I**	**se**	**Model II**	**se**	**Model III**	**se**
Altruistic	0.33***	(0.10)	0.52****	(0.10)	0.43****	(0.10)
Age	0.05****	(0.00)	0.04****	(0.00)	0.03****	(0.00)
Female	0.75****	(0.09)	0.64****	(0.09)	0.58****	(0.08)
**Country**						
DE (base)						
UK	1.22****	(0.21)	1.17****	(0.21)	1.05****	(0.21)
DK	0.60***	(0.22)	0.44**	(0.22)	0.38*	(0.22)
NL	−0.48	(0.32)	−0.70**	(0.31)	−0.72**	(0.31)
FR	−1.25**	(0.58)	−1.57***	(0.57)	−1.77***	(0.57)
PT	1.37****	(0.24)	1.37****	(0.23)	1.16****	(0.23)
IT	−1.18****	(0.17)	−1.11****	(0.17)	−1.18****	(0.16)
**Education**						
Low (base)						
Medium	0.45****	(0.12)	0.41****	(0.12)	0.40****	(0.12)
High	0.39***	(0.13)	0.36***	(0.13)	0.34***	(0.13)
**Income (make end meet)**						
With great difficulty (base)						
With some difficulty	0.19	(0.18)	0.12	(0.18)	0.21	(0.18)
Fairly easily	0.39**	(0.18)	0.28	(0.18)	0.45***	(0.18)
Easily	0.43**	(0.20)	0.27	(0.20)	0.50**	(0.20)
Confirmed cases (cpm)	0.01**	(0.01)	0.02***	(0.01)	0.01***	(0.01)
General risk aversion			0.50****	(0.04)	0.50****	(0.04)
**COVID related risk factors**						
Risk of infection					−0.21**	(0.11)
Risk to own health					0.58****	(0.10)
Risk family health					0.77****	(0.10)
Risk community health					0.30***	(0.09)
Intercept	15.39****	(0.31)	14.41****	(0.31)	14.00****	(0.31)
Observations	5,812		5,812		5,812	
R-squared	0.12		0.15		0.18	

*Robust standard errors in parentheses*.

^****^p < 0.001, ^***^p < 0.01, ^**^p < 0.05, ^*^p < 0.10.

We find a significant positive relationship between being altruistic and a higher score of pro-social behavior in the base model (*p* < 0.01) as well as when we control for risk aversion in Model II (*p* < 0.001) and for COVID-related risk assessments in Model III (*p* < 0.001).

We proceeded to Test Model III with the alternative specifications for altruism (Appendix Table 6). We found a marginally significant effect when altruism is defined as a continuous measure (*p* < 0.10), as in the percentage share donated. When we test the ordinal measure, we find a significant positive association between higher altruism/donation levels and a higher iPSB score. In Appendix Table 13 we furthermore tested Model III with the iPSB by country and find significant positive associations between altruism and the iPSB in the Netherlands (*p* < 0.001), Germany (*p* < 0.05), Italy (*p* < 0.05) and Portugal (*p* < 0.10) for the binary measure. As a further sensitivity check for the iPSB we compare the results of Model 3 using the iPSB and a score derived by employing pca in Appendix Table 14 and find no differences in results.

#### Days of Quarantine After Visiting a High-Risk Area

On average, respondents were willing to spend 9.6 (sd = 4.7) days in quarantine without symptoms (range 0–14) after visiting a high-risk area and taking no test. The duration of a voluntary stay under quarantine was found to be longer among the altruistic as compared to non-altruistic respondents. When analyzing the factors that influence the number of days, we observe a significant effect of altruism (Appendix Table 7). Controlling for sociodemographics and risk assessments, we find that the duration of a voluntary stay under quarantine was still longer among the altruistic than non-altruistic respondents (*p* < 0.000). Other characteristics that were significantly associated with staying at home longer were age (*p* < 0.000), being female (*p* < 0.000), having a middle education (*p* < 0.01) as compared to low (base), and having a very high relative household income (*p* < 0.01). Compared to Germany (base), respondents in five countries were willing to spend more time in quarantine (*p* < 0.000), except in France, where respondents were willing to stay home fewer days (*p* < 0.01). Furthermore, the higher the risk perception of COVID concerning their health (*p* < 0.000), their families' health (*p* < 0.000), their communities' health (*p* < 0.000), and the higher the perceived risk of infection for themselves (*p* < 0.01), the more days respondents would stay at home.

#### Likelihood of Staying at Home (With Symptoms) to Wait for Test Result

On average, 89.4% (sd = 0.3%) of respondents (*N* = 6,732^3^) stated they would be likely or very likely to stay at home for up to 4 days under quarantine to wait for the results of a (PCR) test because of symptoms. Again, controlling for sociodemographics and risk assessments, we find that altruistic respondents would be more likely (*p* < 0.01 Model III) to wait for a test result (Appendix Table 9) for all three models.

#### Going to a Supermarket With COVID-Related Symptoms

On average, 35% (sd = 0.48) of respondents (*N* = 6,502) stated they would be likely or very likely to go to a supermarket when showing COVID-19 or cold-related symptoms (coughing/fever/feeling tired/sneezing). Controlling for sociodemographics and risk assessments, we find that altruistic respondents would be less likely to go to a supermarket with symptoms (Appendix Table 10, *p* < 0.001).

#### Wearing a Face Mask Where It Is Recommended

On average, 90.3% (sd = 0.30) of respondents (*N* = 6,645) stated they would be likely or very likely to wear a facemask where it is recommended (e.g., at large outside gatherings). We find, consistently throughout Model I-III, that being altruistic is significantly associated with being more likely to wear a face mask voluntarily (Appendix Table 10, *p* < 0.001).

#### Wearing a Face Mask Where It Is Mandatory

On average, 93.4% (sd = 0.25) of respondents (*N* = 6,631) stated they would be likely or very likely to wear a facemask where it is mandatory (e.g., public transport, supermarket). For this behavior, we find no significant effect of being altruistic on the likelihood of wearing a mask when it is mandatory (Appendix Table 11). A larger general risk aversion (*p* < 0.000), perceived risk for the health of the family (*p* < 0.01) and community (*p* < 0.01) were associated with a higher likelihood of wearing a face mask where it is mandatory.

#### Inviting More Than Six Persons to One's Own House for an Indoor Gathering

On average, 28.7% (sd = 0.45) of respondents (*N* = 6,688) stated they would be likely or very likely to invite more than six people to an indoor gathering. We again find no significant effect of being altruistic on the likelihood of engaging in this behavior (Appendix Table 12). As shown in Table 3, there is a variation across the ECOS countries. For example, in September 2020, indoor meetings were not forbidden but highly discouraged in Germany (26). Meanwhile, Italy was (in September 2020) in a phase in-between lockdowns, where meetings with more than six people in their own home were allowed and only forbidden in October 2020 (27), which was after the data collection for this article. In Italy, 39.3% of respondents stated that they would be likely to invite more than six people to their house compared to 29.9% in Germany. Differences in the regulation or situation may be a factor in this difference.

A larger general risk aversion (*p* < 0.000), as well as a higher perceived risk of COVID to the own health (*p* < 0.000) and the families' health (*p* < 0.000), was associated with a lower likelihood of hosting such a gathering. Counterintuitively, a perceived higher risk of infection was associated with an increased likelihood of hosting such a gathering (*p* < 0.05). The latter may express people expecting a mild course of the disease if they get infected.

#### Quantitative Magnitude Coefficients

The effect size between the altruistic and non-altruistic group of respondents for six individual behaviors and the iPSB was found to be below Cohen's (28) convention for a small effect size (d ≥ 0.20). Only the days of quarantine after visiting a high-risk country (d = −0.27) exceeded the convention for a small effect size. The effect size for gender for the iPSB (d = −0.13) as well as for all individual behaviors was also below the threshold. We find a similar picture when looking at the iPSB and education, where neither a high (d = −0.08), middle (d = 0.00), nor low level (d = 0.11) would pass the threshold. When looking at the age categories, we find small effects for the iPSB as well as for inviting more than six people to their own house. Respondents between the age of 18–24 stated to be significantly more likely to invite more than six people to their home (M = 2.58, sd = 1.42) compared to other age groups (M = 2.08, sd = 1.33); *t*_(7, 023)_ = [−9.21], *p* < 0.000, using a paired-samples *t*-test, while satisfying the criteria for a small effect size (d = −0.38). We find a similar picture for respondents between the age of 25–34 (d = −0.25). On the other side of the age spectrum, we find that respondents age 65 and above would be less likely to invite more than six people to their house (M = 1.88, sd = 1.21) compared to other age groups (M = 2.19, sd = 1.37); *t*_(7, 023)_ = [8.03], *p* < 0.000 using the same test, also resulting in a small effect size (d = 0.23) when using Cohen's test. Similarly, we find a small effect (d = 0.31) for the iPSB score when comparing 18–24 year-old respondents (M = 18.56, sd = 3.42) to all other age groups (M = 19.64, sd = 3.46); *t*_(5, 996)_ = [6.92], *p* < 0.000 similar to 25–34 (d = 0.28) year-old respondents. Respondents age 55–64 (d = −0.22) and above 65 (d = −0.25) had a slightly higher iPSB score (M = 20.21, sd = 3.13) compared to other age groups (M = 19.35, sd = 3.53); *t*_(5, 996)_ = [−7.96].

## Discussion

Using data from the ECOS study, we investigated if there is a relationship between an overall altruistic disposition (i.e., a concern for others as opposed to self-interest) and behaviors in the COVID-19 pandemic that mainly benefits others. We indeed find such a relationship and can conclude that people who show at least a degree of altruistic behavior will also be more likely to act in a pro-social manner, as expressed in a higher score on our index of pro-social behaviors. The level of significance is higher once we control for risk aversion. This may have to do with the fact that altruistic people are less risk averse in our sample, and risk aversion has been shown to be correlated to positive reciprocity by Falk et al. (29), which in turn is positively related to altruism.

We find a similar result when analyzing the relationship between altruism and individual behaviors. For example, we find that being altruistic is associated with a higher likelihood of not going to a supermarket with COVID-19 symptoms. This is inconvenient or associated with extra cost for the individual (e.g., food delivery services) but contributes to a low infection risk environment for others. This result is intuitive, in line with previous findings [e.g., (8, 9)] and confirms earlier observations during the pandemic (13–15). For two of the behaviors, namely wearing a mask where it is mandatory and inviting more than six people to their own house, we find no significant effect of being altruistic. Different reasons may be the cause for this; more altruistic people could also be more sociable and therefore inclined to invite people. Maybe altruism plays a role in voluntary activities that protect others but not in observing regulations like mandatory mask-wearing. For these regulations, peer pressure and sanctions may play a more important role (10, 30), or it may be driven by individual risk perception and cognitive biases (12).

We further find that general risk aversion plays a role in the pandemic behaviors and the subjective assessment of what risk COVID-19 poses to the individual or the people around her/him. For example, perceiving COVID-19 as a higher risk for one's own health or the health of the family was associated with a higher likelihood of wearing a face mask where it is recommended. This is consistent with other recent findings (31–34) that conclude a higher perceived risk of COVID-19 increases the adoption of preventative measures.

When studying the altruism coefficients in the iPSB model country-by-country (Appendix Table 13), we found relatively large differences. Although there is a lot of noise in this comparison due to the much smaller sample sizes, it is worth speculating about these differences. For example, the coefficients are especially low for the Netherlands, where the government relied a lot on moral appeals with respect to preventive measures at the start of the pandemic. Altruistic Dutch citizens may have reacted stronger to this appeal than non-altruistic Dutch citizens. Governments of other countries have relied less on such moral appeals, and implemented more strict measures, leaving less room for heterogeneity in pro-social behavior among different types of altruists. Future research is encouraged to study the effects of different COVID-related policy measures on the mediating role of altruism on pro-social behavior in more detail.

Some limitations apply; first, our elicitation of altruism comes from economic theory and contrasts it with self-interest. When we tested other definitions of altruism, such as an ordinal measure, we found a significant association between higher donations and pro-social behavior, suggesting that there may be a positive relationship between the amount donated and pro-social behaviors. On the other hand the results of the ordered measure suggest a non-monotonic relationship, which could in turn mean a that the amount does not matter in a hypothetical donation. Of course, altruism, like many behaviors, is better identified by observing behavior (e.g., in experiments) than by eliciting it hypothetically. Furthermore, there are validated questionnaires in psychological research that aim to identify more altruistic people, e.g., by using the simplified SRA scale to assess altruism (35). Future research could compare the results of our measure and other ways of identifying altruistic individuals in stated choice contexts as well as other measures associated with pro-social behavior, such as time preferences, we were not able to control for.

Second, we use stated choice for pandemic behaviors. This always involves the risk of respondents giving socially acceptable or desirable answers. While this is a limitation, we are confident that the anonymity of our questionnaire minimized the risk of socially desirable answers.

Third, related to this, we find only a small change in the size of the donations between the altruistic and non-altruistic group. This could be related to the hypothetical nature of the questions and the donation measure since this is not a behavioral experiment, but a study based on stated choices.

Our results indicate that altruism or regard for the wellbeing of others positively contributes to pandemic behaviors, which in turn contributes to a low infection risk environment. Furthermore, we find that the subjective risk assessment of COVID-19 also plays a role in these behaviors. Especially for risks for which individuals have limited reference points, such as COVID-19 or previous new viruses like H1N1 (swine flu), subjective risk perceptions may play an important role in engaging in protective or avoidance behaviors (36). In order to update these reference points and correct the subjective risk assessments, Bish and Michie (37) suggest using tailored interventions and communication strategies that focus on particular demographic groups to update their perceived threat of the pandemic and the effectiveness of certain protective behaviors. Policymakers could draw from these lessons for the current and possibly future pandemics to improve adherence to and acceptability of measures. In line with earlier findings (10, 11), our results suggest that emphasizing how a particular behavior (e.g., wearing a mask) will protect vulnerable people around us may increase the adherence to the behavior. Furthermore, providing accurate and straightforward information about the infection risks that behaviors may cause (e.g., going to the supermarket with symptoms), could further improve adherence to pro-social behaviors.

## Data Availability Statement

The raw data supporting the conclusions of this article will be made available by the authors, upon reasonable request.

## Ethics Statement

The studies involving human participants were reviewed and approved by University of Hamburg WiSo Laboratories. The patients/participants provided their written informed consent to participate in this study.

## Ecos Consortium

Iryna Sabat, Sebastian Neumann-Böhme, Pedro P. Barros, Werner Brouwer, Job van Exel, Jonas Schreyögg, Tom Stargardt, and Aleksandra Torbica.

## Author Contributions

AA, IS, and SN-B conceived of the presented idea. IS and SN-B programmed the survey and collected the data. SN-B wrote the first draft. All authors contributed to the final draft of the manuscript.

## Funding

This project received funding from the Horizon 2020 Research and Innovation Programme of the European Union through the Marie Skłodowska-Curie Grant Agreement No. 721402 and was further funded by the German Research Foundation (DFG) funds from the Excellence Strategy of the University of Hamburg and other participating universities.

## Conflict of Interest

The authors declare that the research was conducted in the absence of any commercial or financial relationships that could be construed as a potential conflict of interest.

## Publisher's Note

All claims expressed in this article are solely those of the authors and do not necessarily represent those of their affiliated organizations, or those of the publisher, the editors and the reviewers. Any product that may be evaluated in this article, or claim that may be made by its manufacturer, is not guaranteed or endorsed by the publisher.

## Footnotes

^1^Behavior 2. “Go to the supermarket with symptoms” and 3. “Invite more than 6 people to your house” were recoded to match the others so that 3–4 is pro-social and 1–2 is not.

^2^Lottery A: 50/50 chance to double income or to reduce it by 1/3. Lottery B: 50/50 chance to double income or to reduce it by 1/2 (if respondent is willing to take lottery A). Lottery C: 50/50 chance to double income or to reduce it by 1/5 (if respondent is not willing to take lottery A).

^3^As pointed out in the methods, respondents who stated “do not know” were recoded as missing; therefore, the number of respondents varies by behaviour.
