# Supplementary material for: Altruism and the Link to Pro-social Pandemic Behavior
Source: Front Health Serv. 2022 Jul 14;2:871891. doi: 10.3389/frhs.2022.871891 (PMC10012788; doi:10.3389/frhs.2022.871891)
Supplement: Supplementary file 1 [file Data_Sheet_1.docx]

Appendix:

Table 7: Alternative specifications of Altruism

| iPSB | Base | se | Continuous | se | Ordinal | se |
| --- | --- | --- | --- | --- | --- | --- |
| Age | 0.03**** | (0.00) | 0.03**** | (0.00) | 0.03**** | (0.00) |
| Female | 0.44**** | (0.07) | 0.45**** | (0.07) | 0.44**** | (0.07) |
| **Country** |  |  |  |  |  |  |
| DE (base) |  |  |  |  |  |  |
| UK | 0.65**** | (0.18) | 0.63**** | (0.18) | 0.63**** | (0.18) |
| DK | 0.13 | (0.18) | 0.07 | (0.18) | 0.12 | (0.18) |
| NL | -0.62** | (0.26) | -0.66** | (0.26) | -0.63** | (0.26) |
| FR | -1.10** | (0.48) | -1.12** | (0.48) | -1.12** | (0.48) |
| PT | 0.73**** | (0.20) | 0.74**** | (0.20) | 0.73**** | (0.20) |
| IT | -1.59**** | (0.14) | -1.57**** | (0.14) | -1.63**** | (0.14) |
| **Education** |  |  |  |  |  |  |
| Low (base) |  |  |  |  |  |  |
| Medium | 0.33*** | (0.10) | 0.33*** | (0.10) | 0.33*** | (0.10) |
| High | 0.34*** | (0.11) | 0.34*** | (0.11) | 0.34*** | (0.11) |
| **Income (make ends meet)**  With great difficulty (base) |  |  |  |  |  |  |
| With some difficulty | 0.17 | (0.15) | 0.17 | (0.15) | 0.16 | (0.15) |
| Fairly easily | 0.40*** | (0.15) | 0.43*** | (0.15) | 0.40*** | (0.15) |
| Easily | 0.38** | (0.17) | 0.40** | (0.17) | 0.38** | (0.17) |
| Confirmed cases (cpm) | 0.01** | (0.00) | 0.01** | (0.00) | 0.01** | (0.00) |
| General risk aversion | 0.47**** | (0.03) | 0.46**** | (0.03) | 0.47**** | (0.03) |
| **Covid related risk factors** |  |  |  |  |  |  |
| Risk of Infection | -0.31**** | (0.09) | -0.30*** | (0.09) | -0.32**** | (0.09) |
| Risk to own health | 0.41**** | (0.09) | 0.41**** | (0.09) | 0.40**** | (0.09) |
| Risk family health | 0.63**** | (0.08) | 0.63**** | (0.08) | 0.63**** | (0.08) |
| Risk community health | 0.17** | (0.08) | 0.18** | (0.08) | 0.17** | (0.08) |
|  |  |  |  |  |  |  |
| **Altruistic** | 0.25*** | (0.08) |  |  |  |  |
| Alt. Continuous |  |  | -0.00 | (0.00) |  |  |
| Level of Alt. |  |  |  |  |  |  |
| No altruism (base) |  |  |  |  |  |  |
| Low |  |  |  |  | 0.16 | (0.10) |
| Medium |  |  |  |  | 0.39**** | (0.11) |
| High |  |  |  |  | 0.23** | (0.10) |
|  |  |  |  |  |  |  |
|  |  |  |  |  |  |  |
| Constant | 12.13**** | (0.26) | 12.31**** | (0.26) | 12.14**** | (0.26) |
|  |  |  |  |  |  |  |
| Observations | 5,812 |  | 5,812 |  | 5,812 |  |
| R-squared | 0.19 |  | 0.19 |  | 0.19 |  |

Robust standard errors in parentheses

**** p<0.001, *** p<0.01, ** p<0.05, * p<0.10

Table 8: Days of quarantine after visiting a high-risk country without test (OLS)

| Days in Quarantine | Coefficient | Std. error | t | p-value | 95% CI | |  |
| --- | --- | --- | --- | --- | --- | --- | --- |
| Age | 0.017**** | 0.004 | 4.462 | 0.000 | 0.009 | 0.024 | |
| Female | 0.673**** | 0.109 | 6.158 | 0.000 | 0.459 | 0.887 | |
| **Country**  Germany (base) | 0.000 | 0.000 |  |  |  |  | |
| United Kingdom | 2.015**** | 0.196 | 10.257 | 0.000 | 1.630 | 2.400 | |
| Denmark | 1.552**** | 0.211 | 7.359 | 0.000 | 1.139 | 1.966 | |
| Netherlands | 0.695**** | 0.193 | 3.591 | 0.000 | 0.315 | 1.074 | |
| France | -0.622*** | 0.206 | -3.016 | 0.003 | -1.027 | -0.218 | |
| Portugal | 2.420**** | 0.204 | 11.886 | 0.000 | 2.021 | 2.819 | |
| Italy | 1.921**** | 0.201 | 9.553 | 0.000 | 1.527 | 2.315 | |
| **Education**  Low (base) | 0.000 | 0.000 |  |  |  |  | |
| Middle | 0.429*** | 0.151 | 2.836 | 0.005 | 0.132 | 0.725 | |
| High | 0.200 | 0.161 | 1.242 | 0.214 | -0.116 | 0.516 | |
| **Income (make ends meet)**  With great difficulty (base) | 0.000 | 0.000 |  |  |  |  | |
| With some difficulty | 0.296 | 0.223 | 1.326 | 0.185 | -0.141 | 0.733 | |
| Fairly easily | 0.328 | 0.223 | 1.474 | 0.141 | -0.108 | 0.764 | |
| Easily | 0.679*** | 0.254 | 2.677 | 0.007 | 0.182 | 1.176 | |
| **Covid related risk factors**  Risk of Infection | 0.254*** | 0.080 | 3.182 | 0.001 | 0.097 | 0.410 | |
| Risk to own health | 0.362**** | 0.071 | 5.074 | 0.000 | 0.222 | 0.502 | |
| Risk family health | 0.350**** | 0.071 | 4.926 | 0.000 | 0.211 | 0.490 | |
| Risk community health | 0.402**** | 0.076 | 5.262 | 0.000 | 0.252 | 0.552 | |
| General risk aversion | 0.123*** | 0.044 | 2.803 | 0.005 | 0.037 | 0.209 | |
| Altruistic | 0.820**** | 0.131 | 6.272 | 0.000 | 0.564 | 1.076 | |
| Intercept | 1.550**** | 0.414 | 3.741 | 0.000 | 0.738 | 2.362 | |
|  | **** p<.001, *** p<.01, ** p<.05, * p<.1 | | | | | | |

Table 9: Logistic regression results for the likelihood of waiting for a test result at home

|  | Model I |  | Model II |  | Model III |  |
| --- | --- | --- | --- | --- | --- | --- |
| Waiting for test result | Odds ratio | SE | Odds ratio | SE | Odds ratio | SE |
| Age | 1.02**** | (0.00) | 1.01**** | (0.00) | 1.02**** | (0.00) |
| Female | 1.29*** | (0.11) | 1.23** | (0.10) | 1.21** | (0.10) |
| **Country** |  |  |  |  |  |  |
| Germany (base) |  |  |  |  |  |  |
| United Kingdom | 1.54** | (0.32) | 1.49* | (0.31) | 1.43* | (0.30) |
| Denmark | 1.81*** | (0.40) | 1.68** | (0.37) | 1.63** | (0.36) |
| Netherlands | 1.25 | (0.37) | 1.13 | (0.33) | 1.13 | (0.33) |
| France | 0.86 | (0.47) | 0.74 | (0.41) | 0.69 | (0.38) |
| Portugal | 1.67** | (0.38) | 1.66** | (0.38) | 1.53* | (0.36) |
| Italy | 1.26 | (0.20) | 1.28 | (0.21) | 1.24 | (0.20) |
| **Education** |  |  |  |  |  |  |
| Low (base) |  |  |  |  |  |  |
| Middle | 1.45**** | (0.16) | 1.42*** | (0.15) | 1.42*** | (0.15) |
| High | 1.40*** | (0.17) | 1.39*** | (0.17) | 1.39*** | (0.17) |
| **Income (make ends meet)** |  |  |  |  |  |  |
| Great difficulty (base) |  |  |  |  |  |  |
| With some difficulty | 1.23 | (0.17) | 1.20 | (0.17) | 1.22 | (0.17) |
| Fairly easily | 1.53*** | (0.22) | 1.46*** | (0.21) | 1.52*** | (0.22) |
| Easily | 1.40* | (0.24) | 1.31 | (0.23) | 1.39* | (0.24) |
|  |  |  |  |  |  |  |
| Confirmed cases (cpm) | 1.00 | (0.01) | 1.00 | (0.01) | 1.00 | (0.01) |
| General risk aversion |  |  | 1.23**** | (0.04) | 1.23**** | (0.04) |
| **Covid related risk factors** |  |  |  |  |  |  |
| Risk of Infection |  |  |  |  | 0.83* | (0.09) |
| Risk to own health |  |  |  |  | 1.11 | (0.13) |
| Risk family health |  |  |  |  | 1.46**** | (0.15) |
| Risk community health |  |  |  |  | 1.17 | (0.12) |
|  |  |  |  |  |  |  |
| Altruistic | 1.23** | (0.11) | 1.35**** | (0.12) | 1.30*** | (0.12) |
|  |  |  |  |  |  |  |
| Constant | 1.29 | (0.33) | 0.87 | (0.23) | 0.75 | (0.21) |
|  |  |  |  |  |  |  |
| Observations | 6,522 |  | 6,522 |  | 6,522 |  |
| Pseudo R-squared | 0.030 |  | 0.039 |  | 0.046 |  |

Robust SE in parentheses

**** p<0.001, *** p<0.01, ** p<0.05, * p<0.10

Table 10: Logistic regression results for the likelihood of going to the supermarket with symptoms

| Going to supermarket with symptoms | Model I |  | Model II |  | Model III |  |
| --- | --- | --- | --- | --- | --- | --- |
|  | Odds ratio | SE | Odds ratio | SE | Odds ratio | SE |
| Age | 0.98**** | (0.00) | 0.98**** | (0.00) | 0.98**** | (0.00) |
| Female | 0.67**** | (0.04) | 0.70**** | (0.04) | 0.72**** | (0.04) |
| **Country** |  |  |  |  |  |  |
| Germany (base) |  |  |  |  |  |  |
| United Kingdom | 0.80* | (0.11) | 0.83 | (0.11) | 0.85 | (0.12) |
| Denmark | 0.76* | (0.11) | 0.83 | (0.12) | 0.84 | (0.12) |
| Netherlands | 1.18 | (0.23) | 1.34 | (0.26) | 1.34 | (0.26) |
| France | 2.15** | (0.80) | 2.64** | (1.00) | 2.70*** | (1.03) |
| Portugal | 0.60*** | (0.10) | 0.60*** | (0.10) | 0.63*** | (0.10) |
| Italy | 1.34*** | (0.14) | 1.30** | (0.14) | 1.35*** | (0.14) |
| **Education** |  |  |  |  |  |  |
| Low (base) |  |  |  |  |  |  |
| Middle | 1.05 | (0.08) | 1.08 | (0.08) | 1.09 | (0.08) |
| High | 1.15* | (0.09) | 1.16* | (0.09) | 1.17* | (0.10) |
| **Income (make ends meet)** |  |  |  |  |  |  |
| Great difficulty (base) |  |  |  |  |  |  |
| With some difficulty | 0.94 | (0.10) | 0.98 | (0.10) | 0.95 | (0.10) |
| Fairly easily | 0.86 | (0.09) | 0.92 | (0.10) | 0.89 | (0.09) |
| Easily | 0.87 | (0.10) | 0.96 | (0.12) | 0.90 | (0.11) |
|  |  |  |  |  |  |  |
| Confirmed cases (cpm) | 0.99 | (0.00) | 0.99** | (0.00) | 0.99* | (0.00) |
| General risk aversion |  |  | 0.76**** | (0.02) | 0.76**** | (0.02) |
| **Covid related risk factors** |  |  |  |  |  |  |
| Risk of Infection |  |  |  |  | 1.44**** | (0.11) |
| Risk to own health |  |  |  |  | 0.80*** | (0.06) |
| Risk family health |  |  |  |  | 0.76**** | (0.05) |
| Risk community health |  |  |  |  | 0.87* | (0.06) |
|  |  |  |  |  |  |  |
| Altruistic | 0.87** | (0.05) | 0.77**** | (0.05) | 0.78**** | (0.05) |
|  |  |  |  |  |  |  |
| Constant | 2.66**** | (0.50) | 4.63**** | (0.91) | 4.94**** | (0.99) |
|  |  |  |  |  |  |  |
| Observations | 6,297 |  | 6,297 |  | 6,297 |  |
| Pseudo R-squared | 0.039 |  | 0.057 |  | 0.064 |  |

Robust SE in parentheses

**** p<0.001, *** p<0.01, ** p<0.05, * p<0.10

Table 11: Logistic regression results for the likelihood of wearing a face mask where recommended

| Wear mask where it is recommended | Model I |  | Model II |  | Model III |  |
| --- | --- | --- | --- | --- | --- | --- |
|  | Odds ratio | SE | Odds ratio | SE | Odds ratio | SE |
| Age | 1.02**** | (0.00) | 1.02**** | (0.00) | 1.02**** | (0.00) |
| Female | 1.30*** | (0.12) | 1.27*** | (0.11) | 1.24** | (0.11) |
| **Country** |  |  |  |  |  |  |
| Germany (base) |  |  |  |  |  |  |
| United Kingdom | 1.39 | (0.29) | 1.35 | (0.28) | 1.30 | (0.27) |
| Denmark | 0.98 | (0.21) | 0.93 | (0.20) | 0.91 | (0.19) |
| Netherlands | 0.52** | (0.15) | 0.48** | (0.14) | 0.49** | (0.14) |
| France | 0.65 | (0.36) | 0.58 | (0.32) | 0.55 | (0.31) |
| Portugal | 1.89*** | (0.46) | 1.87** | (0.46) | 1.72** | (0.43) |
| Italy | 1.57*** | (0.27) | 1.59*** | (0.27) | 1.57*** | (0.27) |
| **Education** |  |  |  |  |  |  |
| Low (base) |  |  |  |  |  |  |
| Middle | 1.45**** | (0.16) | 1.44*** | (0.16) | 1.44*** | (0.16) |
| High | 1.44*** | (0.18) | 1.43*** | (0.18) | 1.43*** | (0.18) |
| **Income (make ends meet)** |  |  |  |  |  |  |
| Great difficulty (base) |  |  |  |  |  |  |
| With some difficulty | 1.37** | (0.20) | 1.35** | (0.19) | 1.42** | (0.21) |
| Fairly easily | 1.71**** | (0.25) | 1.66**** | (0.25) | 1.85**** | (0.28) |
| Easily | 1.41** | (0.24) | 1.36* | (0.23) | 1.55** | (0.27) |
|  |  |  |  |  |  |  |
| Confirmed cases (cpm) | 1.01 | (0.01) | 1.01* | (0.01) | 1.01 | (0.01) |
| General risk aversion |  |  | 1.14**** | (0.04) | 1.15**** | (0.04) |
| **Covid related risk factors** |  |  |  |  |  |  |
| Risk of Infection |  |  |  |  | 0.87 | (0.11) |
| Risk to own health |  |  |  |  | 1.64**** | (0.20) |
| Risk family health |  |  |  |  | 1.50**** | (0.17) |
| Risk community health |  |  |  |  | 1.09 | (0.12) |
|  |  |  |  |  |  |  |
| Altruistic | 1.47**** | (0.13) | 1.56**** | (0.14) | 1.48**** | (0.14) |
|  |  |  |  |  |  |  |
| Constant | 0.85 | (0.23) | 0.65 | (0.18) | 0.54** | (0.16) |
|  |  |  |  |  |  |  |
| Observations | 6,437 |  | 6,437 |  | 6,437 |  |
| Pseudo R-squared | 0.043 |  | 0.046 |  | 0.061 |  |

Robust SE in parentheses

**** p<0.001, *** p<0.01, ** p<0.05, * p<0.10

Table 12: Logistic regression results for the likelihood of wearing a face mask where mandatory

| Wear mask where it is mandatory | Model I |  | Model II |  | Model III |  |
| --- | --- | --- | --- | --- | --- | --- |
|  | Odds ratio | SE | Odds ratio | SE | Odds ratio | SE |
| Age | 1.02**** | (0.00) | 1.02**** | (0.00) | 1.02**** | (0.00) |
| Female | 1.47**** | (0.15) | 1.39*** | (0.15) | 1.34*** | (0.14) |
| **Country** |  |  |  |  |  |  |
| Germany (base) |  |  |  |  |  |  |
| United Kingdom | 1.62* | (0.41) | 1.54* | (0.39) | 1.47 | (0.38) |
| Denmark | 1.45 | (0.38) | 1.31 | (0.35) | 1.27 | (0.34) |
| Netherlands | 0.97 | (0.34) | 0.83 | (0.30) | 0.84 | (0.30) |
| France | 1.54 | (1.07) | 1.24 | (0.87) | 1.19 | (0.84) |
| Portugal | 1.94** | (0.56) | 1.89** | (0.55) | 1.72* | (0.50) |
| Italy | 1.21 | (0.23) | 1.23 | (0.23) | 1.18 | (0.22) |
| **Education** |  |  |  |  |  |  |
| Low (base) |  |  |  |  |  |  |
| Middle | 1.68**** | (0.22) | 1.64**** | (0.21) | 1.62**** | (0.21) |
| High | 1.68**** | (0.24) | 1.67**** | (0.24) | 1.66**** | (0.24) |
| **Income (make end meet)** |  |  |  |  |  |  |
| Great difficulty (base) |  |  |  |  |  |  |
| With some difficulty | 1.92**** | (0.30) | 1.87**** | (0.29) | 1.93**** | (0.30) |
| Fairly easily | 2.51**** | (0.40) | 2.38**** | (0.38) | 2.54**** | (0.41) |
| Easily | 2.23**** | (0.45) | 2.07**** | (0.42) | 2.26**** | (0.46) |
|  |  |  |  |  |  |  |
| Confirmed cases (cpm) | 1.00 | (0.01) | 1.00 | (0.01) | 1.00 | (0.01) |
| General risk aversion |  |  | 1.26**** | (0.05) | 1.26**** | (0.05) |
| **Covid related risk factors** |  |  |  |  |  |  |
| Risk of Infection |  |  |  |  | 0.69*** | (0.10) |
| Risk to own health |  |  |  |  | 1.29* | (0.19) |
| Risk family health |  |  |  |  | 1.76**** | (0.23) |
| Risk community health |  |  |  |  | 1.11 | (0.14) |
|  |  |  |  |  |  |  |
| Altruistic | 1.01 | (0.11) | 1.13 | (0.13) | 1.08 | (0.13) |
|  |  |  |  |  |  |  |
| Constant | 0.96 | (0.30) | 0.61 | (0.20) | 0.53* | (0.18) |
|  |  |  |  |  |  |  |
| Observations | 6,425 |  | 6,425 |  | 6,425 |  |
| Pseudo R-squared | 0.041 |  | 0.051 |  | 0.063 |  |

Robust SE in parentheses

**** p<0.001, *** p<0.01, ** p<0.05, * p<0.10

Table 13: Logistic regression results for the likelihood of inviting more than 6 people to the own house

|  | Model I |  | Model II |  | Model III |  |
| --- | --- | --- | --- | --- | --- | --- |
| Invite > 6 people to house | Odds ratio | SE | Odds ratio | SE | Odds ratio | SE |
| Age | 0.97**** | (0.00) | 0.97**** | (0.00) | 0.98**** | (0.00) |
| Female | 0.74**** | (0.04) | 0.79**** | (0.05) | 0.80**** | (0.05) |
| **Country** |  |  |  |  |  |  |
| Germany (base) |  |  |  |  |  |  |
| United Kingdom | 0.64*** | (0.09) | 0.66*** | (0.09) | 0.66*** | (0.10) |
| Denmark | 1.22 | (0.18) | 1.38** | (0.21) | 1.41** | (0.21) |
| Netherlands | 1.03 | (0.21) | 1.22 | (0.25) | 1.21 | (0.25) |
| France | 2.01* | (0.78) | 2.62** | (1.04) | 2.60** | (1.04) |
| Portugal | 0.73* | (0.12) | 0.74* | (0.12) | 0.77 | (0.13) |
| Italy | 1.54**** | (0.17) | 1.51**** | (0.17) | 1.51**** | (0.17) |
| **Education** |  |  |  |  |  |  |
| Low (base) |  |  |  |  |  |  |
| Middle | 0.96 | (0.08) | 0.98 | (0.08) | 1.00 | (0.08) |
| High | 0.95 | (0.08) | 0.95 | (0.08) | 0.95 | (0.08) |
| **Income (make ends meet)** |  |  |  |  |  |  |
| Great difficulty (base) |  |  |  |  |  |  |
| With some difficulty | 0.97 | (0.10) | 1.02 | (0.11) | 1.00 | (0.11) |
| Fairly easily | 0.98 | (0.11) | 1.06 | (0.12) | 1.03 | (0.11) |
| Easily | 1.01 | (0.13) | 1.13 | (0.14) | 1.08 | (0.14) |
|  |  |  |  |  |  |  |
| Confirmed cases (cpm) | 0.99 | (0.00) | 0.99** | (0.00) | 0.99** | (0.00) |
| General risk aversion |  |  | 0.74**** | (0.02) | 0.74**** | (0.02) |
| **Covid related risk factors** |  |  |  |  |  |  |
| Risk of Infection |  |  |  |  | 1.30**** | (0.10) |
| Risk to own health |  |  |  |  | 0.77**** | (0.06) |
| Risk family health |  |  |  |  | 0.72**** | (0.05) |
| Risk community health |  |  |  |  | 1.15* | (0.08) |
|  |  |  |  |  |  |  |
| Altruistic | 1.02 | (0.07) | 0.89* | (0.06) | 0.90 | (0.06) |
|  |  |  |  |  |  |  |
| Constant | 2.56**** | (0.49) | 4.81**** | (0.98) | 5.01**** | (1.04) |
|  |  |  |  |  |  |  |
| Observations | 6,481 |  | 6,481 |  | 6,481 |  |
| Pseudo R-squared | 0.052 |  | 0.074 |  | 0.079 |  |

Robust SE in parentheses

**** p<0.001, *** p<0.01, ** p<0.05, * p<0.10

Table 14: By country results of iPSB full model

| iPSB | DE | UK | DK | NL | FR | PT | IT |
| --- | --- | --- | --- | --- | --- | --- | --- |
| Age | 0.05**** | 0.04**** | 0.03**** | 0.04**** | 0.03**** | 0.02**** | 0.01 |
| Female | 0.28 | 0.65**** | 0.67**** | 0.84**** | 0.58*** | 0.34* | 0.01 |
| **Education** |  |  |  |  |  |  |  |
| Low (base) |  |  |  |  |  |  |  |
| Medium | 0.91** | 0.47 | 0.30 | 0.51* | 0.17 | 0.34 | 0.05 |
| High | 0.37 | 0.88*** | 0.50 | 0.36 | 0.15 | 0.55** | 0.00 |
| **Income (make end meet)** With great difficulty (base) |  |  |  |  |  |  |  |
| With some difficulty | 0.02 | 1.21** | -0.25 | 0.12 | 0.58* | -0.28 | -0.11 |
| Fairly easily | 0.44 | 1.42*** | 0.02 | 0.47 | 0.31 | 0.22 | 0.05 |
| Easily | 0.65* | 1.28** | -0.27 | 0.37 | 1.08** | 0.04 | -0.21 |
|  |  |  |  |  |  |  |  |
| Confirmed cases (cpm) | -0.08 | -0.00 | 0.01 | 0.01 | 0.02 | 0.00 | 0.28 |
| General risk aversion | 0.51**** | 0.66**** | 0.33**** | 0.72**** | 0.52**** | 0.37**** | 0.18** |
| **Covid related risk factors** |  |  |  |  |  |  |  |
| Risk of Infection | -0.09 | -0.55** | -0.01 | -0.04 | -0.17 | -0.27 | -0.93**** |
| Risk to own health | 0.48* | 0.31 | 0.66**** | 0.54** | 0.56** | -0.02 | 0.42 |
| Risk family health | 0.62*** | 0.71**** | 0.83**** | 0.63*** | 0.61** | 0.49** | 0.61** |
| Risk community health | 0.28 | 0.62*** | 0.22 | 0.51** | -0.04 | 0.21 | -0.57** |
|  |  |  |  |  |  |  |  |
| **Altruistic** | 0.46* | -0.08 | -0.04 | 0.79**** | 0.13 | 0.25 | 0.30 |
|  |  |  |  |  |  |  |  |
| Constant | 12.15**** | 11.07**** | 13.18**** | 9.22**** | 10.13**** | 14.06**** | 7.10 |
|  |  |  |  |  |  |  |  |
| Observations | 825 | 829 | 842 | 815 | 786 | 879 | 836 |
| R-squared | 0.19 | 0.24 | 0.13 | 0.19 | 0.13 | 0.09 | 0.04 |

Robust standard errors in parentheses

**** p<0.001, *** p<0.01, ** p<0.05, * p<0.10

| iPSB | DE | UK | DK | NL | FR | PT | IT |
| --- | --- | --- | --- | --- | --- | --- | --- |
| Age | 0.05**** | 0.04**** | 0.03**** | 0.04**** | 0.03**** | 0.02**** | 0.01 |
| Female | 0.28 | 0.65**** | 0.67**** | 0.86**** | 0.56*** | 0.33* | 0.01 |
| **Education** |  |  |  |  |  |  |  |
| Low (base) |  |  |  |  |  |  |  |
| Medium | 0.92** | 0.47 | 0.30 | 0.50* | 0.15 | 0.34 | 0.05 |
| High | 0.38 | 0.87*** | 0.50 | 0.34 | 0.13 | 0.56** | 0.00 |
| **Income (make end meet)** With great difficulty (base) |  |  |  |  |  |  |  |
| With some difficulty | 0.04 | 1.22** | -0.25 | 0.08 | 0.57 | -0.29 | -0.12 |
| Fairly easily | 0.43 | 1.41*** | 0.02 | 0.46 | 0.33 | 0.20 | 0.05 |
| Easily | 0.62 | 1.30** | -0.27 | 0.35 | 1.08** | 0.01 | -0.21 |
|  |  |  |  |  |  |  |  |
| Confirmed cases (cpm) | -0.08 | -0.00 | 0.01 | 0.01 | 0.02 | 0.01 | 0.28 |
| General risk aversion | 0.52**** | 0.65**** | 0.33**** | 0.72**** | 0.51**** | 0.37**** | 0.18** |
| **Covid related risk factors** |  |  |  |  |  |  |  |
| Risk of Infection | -0.11 | -0.54** | -0.00 | -0.03 | -0.19 | -0.27 | -0.93**** |
| Risk to own health | 0.48* | 0.33 | 0.66**** | 0.54** | 0.58** | -0.03 | 0.42 |
| Risk family health | 0.62** | 0.69**** | 0.83**** | 0.64*** | 0.66** | 0.51*** | 0.61** |
| Risk community health | 0.27 | 0.64**** | 0.22 | 0.52** | -0.08 | 0.20 | -0.57** |
| **Level of Alt.** |  |  |  |  |  |  |  |
| No altruism (base) |  |  |  |  |  |  |  |
| Low | 0.29 | -0.06 | -0.02 | 0.55* | 0.03 | 0.14 | 0.28 |
| Medium | 0.27 | 0.08 | -0.13 | 1.46**** | 0.55** | 0.42 | 0.32 |
| High | 0.60** | -0.28 | 0.01 | 0.65*** | -0.20 | 0.28 | 0.28 |
|  |  |  |  |  |  |  |  |
| Constant | 12.12**** | 11.06**** | 13.18**** | 9.32**** | 10.27**** | 14.05**** | 7.13 |
| Observations | 825 | 829 | 842 | 815 | 786 | 879 | 836 |
| R-squared | 0.19 | 0.24 | 0.13 | 0.20 | 0.14 | 0.10 | 0.04 |

Table 15: By country results of iPSB full model altrenative specification

Robust standard errors in parentheses

**** p<0.001, *** p<0.01, ** p<0.05, * p<0.10
